# Advanced Microfluidic Models of Cancer and Immune Cell Extravasation: A Systematic Review of the Literature

**DOI:** 10.3389/fbioe.2020.00907

**Published:** 2020-08-26

**Authors:** Carlotta Mondadori, Martina Crippa, Matteo Moretti, Christian Candrian, Silvia Lopa, Chiara Arrigoni

**Affiliations:** ^1^IRCCS Istituto Ortopedico Galeazzi, Cell and Tissue Engineering Laboratory, Milan, Italy; ^2^Department of Chemistry, Materials, and Chemical Engineering “Giulio Natta”, Politecnico di Milano, Milan, Italy; ^3^Regenerative Medicine Technologies Laboratory, Ente Ospedaliero Cantonale (EOC), Lugano, Switzerland

**Keywords:** extravasation, microfluidic, cancer cells, immune cells, *in vitro* models

## Abstract

Extravasation is a multi-step process implicated in many physiological and pathological events. This process is essential to get leukocytes to the site of injury or infection but is also one of the main steps in the metastatic cascade in which cancer cells leave the primary tumor and migrate to target sites through the vascular route. In this perspective, extravasation is a double-edged sword. This systematic review analyzes microfluidic 3D models that have been designed to investigate the extravasation of cancer and immune cells. The purpose of this systematic review is to provide an exhaustive summary of the advanced microfluidic 3D models that have been designed to study the extravasation of cancer and immune cells, offering a perspective on the current state-of-the-art. To this end, we set the literature search cross-examining PUBMED and EMBASE databases up to January 2020 and further included non-indexed references reported in relevant reviews. The inclusion criteria were defined in agreement between all the investigators, aimed at identifying studies which investigate the extravasation process of cancer cells and/or leukocytes in microfluidic platforms. Twenty seven studies among 174 examined each step of the extravasation process exploiting 3D microfluidic devices and hence were included in our review. The analysis of the results obtained with the use of microfluidic models allowed highlighting shared features and differences in the extravasation of immune and cancer cells, in view of the setup of a common framework, that could be beneficial for the development of therapeutic approaches fostering or hindering the extravasation process.

## Introduction

Extravasation is the process in which cells that are flowing into a vascular vessel interact with the endothelium lumen, adhere to it, and then cross the endothelial barrier to reach a target site, guided by various types of stimulation. This process represents a key step in several pathologic conditions, for this reason many researchers are focusing on trying to understand and control this phenomenon.

Leukocytes typically extravasate in inflammatory conditions and although the inflammatory response is fundamental to fight infection and in wound healing, the persistency of an active immune response is involved in several pathologies and chronic inflammatory disorders (Schnoor et al., 2015). Extravasation is also crucial during the metastatic cascade, whereby circulating cancer cells deriving from the primary tumor cross the endothelial barrier of specific organs to reach the targeted metastatic site (Reymond et al., 2013).

Extravasation consists in a series of sequential steps that are basically the same for each extravasating cell, and we refer the reader to specific reviews for the exhaustive description of activated pathways during leukocyte (Vestweber, 2015) and cancer cell (Reymond et al., 2013) extravasation. On a more general point of view, extravasation starts with the formation of adhesive interactions between circulating cells and endothelial cells which cover the lumen of the vessels. The process continues with tethering, rolling, and slow-rolling, followed by firm adhesion, crawling, and formation of the transmigratory cup on the endothelial surface. The next step consists in the transendothelial migration that can take place either in a paracellular (crossing the cell endothelial junctions) or in a transcellular (crossing endothelial cells) way. The paracellular way is largely studied due to the relation with the endothelial junction control that seems to be a promising therapeutic target. After passing the endothelial barrier, the extravasating cells must cross the pericyte layer and invade the basement membrane to reach the inflamed tissue or the target secondary organ. Even if the extravasation steps are essentially the same, according to the type of extravasating cells, there are differences in cell responsiveness to specific chemoattractants and diverse activation and/or expression of adhesion molecules mediating cell interactions with the endothelium (Schnoor et al., 2015).

Leukocyte extravasation is usually induced by tissue damage or by infection, which activate the defensive mechanisms of the body. The process starts with the release of proinflammatory cytokines in the damaged tissue, causing endothelial activation (Vestweber, 2012). This activation triggers a cascade of events that enables circulating leukocytes to recognize the vascular endothelium of the inflamed tissue and interact with the endothelial cells. Endothelial selectins (E-selectin, P-selectin) are expressed by the inflamed endothelium and capture leukocytes from the blood flow. Then, chemokines and other chemoattractants produced by endothelial cells and inflammatory cells increase the expression in leukocytes of integrins that bond specific endothelial adhesion molecules (e.g., intracellular adhesion molecule-1, ICAM-1, or vascular adhesion molecules 1, VCAM-1). This bond is essential and leads to leukocyte transendothelial migration (Vestweber, 2015).

Targeting leukocyte extravasation can be a promising approach either for the enhancement of immune defenses or for the suppression of inflammation-induced tissue destruction. For example, anti-adhesion therapies, contrasting self-destructive inflammation, are promising therapeutic options for multiple sclerosis (Vestweber, 2015). On the other hand, studies focusing on the extravasation of cancer cells are always aimed at contrasting this process. Indeed, extravasation is a crucial step of metastasis formation, which leads to 90% of cancer related deaths (Reymond et al., 2013). Cancer cells that intravasate into the blood stream must survive to the aggression of immune cells and to the presence of elevated shear stress, only then they can eventually adhere to the blood vessel wall. Cancer cell extravasation preferentially takes place in small capillaries of the same diameter of the cells, suggesting that the process starts with a physical restriction where the formation of a stable adhesion occurs. The adhesion of cancer cells to the endothelium also requires the expression of ligands and related receptors on both cancer and endothelial cells, including integrins, selectins, cadherins, and immunoglobulin superfamily receptors. It is known that diverse tumors metastasize preferentially in specific tissues/organs, following metastatic patterns that can be related to the particular type of vasculature of the secondary site and to chemokine receptors and complementary chemokines expressed between target endothelium and cancer cells (Nguyen et al., 2009). There are specific chemokines frequently involved in cancer cells extravasation, such as CXC-chemokine ligand 12 (CXCL12), which are secreted by stromal cells placed in distant organs that stimulate cancer cells extravasation and migration to these secondary sites. The paracellular transendothelial migration of cancer cells is usually related to the disruption of endothelial junctions and it is the event that is principally observed *in vitro*. Although there is a small amount of evidence that the cancer transendothelial migration could also be transcellular, this behavior could be related to the characteristic of the vascular bed or the cancer type. This aspect has not yet been fully clarified (Reymond et al., 2013).

The extravasation process has been studied both in *in vivo* and *in vitro* models. Several types of animal models have been used, such as zebrafishes, rats, and mice and, less frequently, dogs for the study of cancer cell and leukocyte extravasation (Simmons et al., 2015; Brown et al., 2017; Gomez-Cuadrado et al., 2017; Marcovecchio et al., 2017). Among all these options, laboratory mice represent the most commonly used animal model, due to their superior physiological and genetic similarities with humans as compared to zebrafishes, but also by their ease of maintenance, breeding and short gestation time as compared to dogs. Moreover, the ability to genetically manipulate mice both by transgenic expression and knockout of specific genes makes mice more versatile for studying human cancer metastasis (Saxena and Christofori, 2013), but also for identifying the role of specific factors in leukocyte trafficking (Power, 2003). Cancer cell extravasation models can be based on the injection of human metastatic cancer cells into immune-compromised animals (xenograft) or on the creation of genetically engineered animals, reproducing the stages of tumor progression (Saxena and Christofori, 2013). In the first case, the use of immunocompromised mice, which is required for experiments using human cancer cells, hinders the possibility to study interactions between cancer and immune cells, that play a critical role in metastasis (Ma et al., 2018). On the other hand, the use of genetically modified mice allows the preservation of the immune system, although these models are not available for all tumor types (Kovar et al., 2016). In both cases, animal immune system, endothelium, and specific tissue-secreted molecules are different from the human ones, possibly altering the observed mechanisms underlying cell behavior (Willyard, 2018). Furthermore, although *in vivo* models allow mimicking the complexity of cell extravasation in a physiological context, they do not permit to investigate how the different elements impact on the phenomenon.

Besides animal models, the study of cancer cell behavior has long been based on standard 2D and 3D *in vitro* cell culture models. The use of these models has allowed investigating many cancer-related events, although standard cell culture models present some critical issues. The principal limitation of 2D models is the lack of the 3D structure of human tissues, which can lead to an abnormal cell behavior. On the other hand, even if classical 3D *in vitro* models have succeeded in mimicking the cancer architecture, they are barely able to reproduce physiological features such as vascularization and blood flow, the presence of biochemical gradients, and the heterogeneity of cell populations that characterize cancer microenvironment (Sleeboom et al., 2018). More specifically, among *in vitro* systems, transwell inserts have been largely used to model the extravasation process. However, although these models allow studying cell adhesion to the endothelium and cell migration through it, they cannot reproduce the extravasation process in dynamic conditions and are not suitable to investigate tissue invasion. Furthermore, extravasation in transwell assays occurs through 2D circular pores measuring from 3 to 12 μm in diameter, which do not match the endothelial junction architecture, and the extravasation can be strongly influenced by gravity force (Kim et al., 2016). Toward a better modeling of the *in vivo* environment some hybrid models have been developed, such as transwell-microfluidic systems that allow including and controlling both luminal and transmural flow (Sleeboom et al., 2018).

Engineered microfluidic devices are promising *in vitro* tools that can overcome the above-mentioned limitations of *in vitro* models in the study of extravasation. The use of microfluidic devices spread out in the last decades thanks to the development of soft lithography. This technological advancement upgraded rapid prototyping allowing the researchers to increase the sophistication and complexity of microfluidic systems (Streets and Huang, 2013). The possibility to easily design and fabricate microfluidic devices also contributed to their versatility, enabling the addition of different features according to the specific microenvironment and phenomenon they are aimed to mimic. Microfluidic devices are all basically constituted by chambers and micro-scale fluidic circuits with a dimension around tens to hundreds of micrometers. The microscale dimension represents an important advantage in biological research, allowing more precise and quantitative measurements, dramatically reducing the number of cells and reagents needed, and hence decreasing also the cost of each experiment (Streets and Huang, 2013). These devices permit to include diverse cellular populations in a 3D microenvironment, allowing to mimic complex physiological microenvironments (Ma et al., 2018). It is also possible to precisely control biophysical and biochemical conditions, and directly visualize in real-time the investigated events. Another key aspect is the possibility to develop systems entirely composed by human cells embedded in 3D extracellular matrices to recapitulate the *in vivo* behavior of cells (Coughlin and Kamm, 2020). In particular, microfluidic models allow including all the main elements involved in the process of extravasation (e.g., geometry of the blood vessel, presence of a 3D environment, etc.) within a cell culture device, thus reproducing the architecture of the *in vivo* milieu. These models can be further implemented to become even more sophisticated by incorporating non-cellular components of the tissue stroma or including multiple types of tissue-specific cells (Coughlin and Kamm, 2020).

Up to now, microfluidic models have been exploited to dissect specific effects of biophysical, biochemical and cellular elements on leukocyte or cancer cell extravasation. In the present systematic review, we will discuss the findings achieved through the use of microfluidic systems, highlighting specific model features which enabled to achieve the reported results.

## Methods

### Search Strategy

The literature search was aimed to identify all the studies describing microfluidic models designed to investigate cancer cell and/or leukocyte extravasation. The literature search was carried out consulting PUBMED and EMBASE databases. We also checked the reference lists of relevant reviews to include other studies that had not been identified during the search process. The full search strategy is reported in Appendix S1.

### Study Selection

Inclusion criteria were defined to select the studies. Specifically, we included studies describing the use of a microfluidic model and investigating the process of cancer cell and/or leukocyte extravasation. Two investigators (CM and MC) independently reviewed the literature and classified the references based on the title and abstract. The eligible articles were further screened through the available full-text, and the studies matching the inclusion criteria were selected. Upon full-text reading, some studies were excluded due to the reasons described in detail in section Study Selection and Features of the Study. Any disagreement on study eligibility was solved by discussion.

### Data Extraction and Analysis

Data extraction was performed by three investigators (CM, MC, and SL). Any disagreement was solved by discussion. The following data were extracted: type of extravasating cells, type of endothelial setting (i.e., endothelial monolayer, endothelial channel, microvascular network), type of biophysical factor(s) applied in the system, type of biochemical factor(s) applied in the system, type of environmental factors present (i.e., presence of tissue-specific cells, features of the extracellular matrix), properties of extravasating cells (i.e., tissue of origin, metastatic potential, cell stiffness). We specifically focused on those factors that can be studied taking advantage of the features of microfluidic extravasation models, with the final aim to illustrate their potential and provide examples of the scientific questions that can be addressed using this type of models.

### Outcome(s)

The primary outcome was the effect of any of the factors mentioned above (biochemical factors, biophysical factors, environmental factors, intrinsic cell properties) on the transendothelial migration of extravasating cells detected in the presence of any of these factors in comparison to a control condition.

The effect of the same factors on extravasation phases that precede or follow the transendothelial migration (i.e., rolling, adhesion, matrix invasion) were considered as secondary outcomes.

### Quality and Risk of Bias Assessment

The methodological quality of the studies and the risk of bias were assessed adapting methodologies described in previous systematic reviews focusing on *in vitro* studies (AlShwaimi et al., 2016; Golbach et al., 2016). Two investigators (CM and SL) performed independently the quality assessment. The following biases were evaluated: (1) study design bias, (2) reporting bias, (3) detection bias. The scoring system included four possible answers to the questions reported in Table 2. If the paper satisfied totally or partially the request, the scores were respectively “Yes” or “Partly.” If the paper did not satisfy the request, the score was “No.” “Unclear” was attributed when the paper lacked the necessary details to assess the risk and, hence, it was not possible to attribute any of the other three answers. The overall quality was then determined as follows: The articles that reported 1–3 “Yes” items were classified as high risk of bias, 4–6 as moderate risk of bias, and 6–9 as low risk of bias.

## Results

### Study Selection and Features of the Study

**Figure 1:**
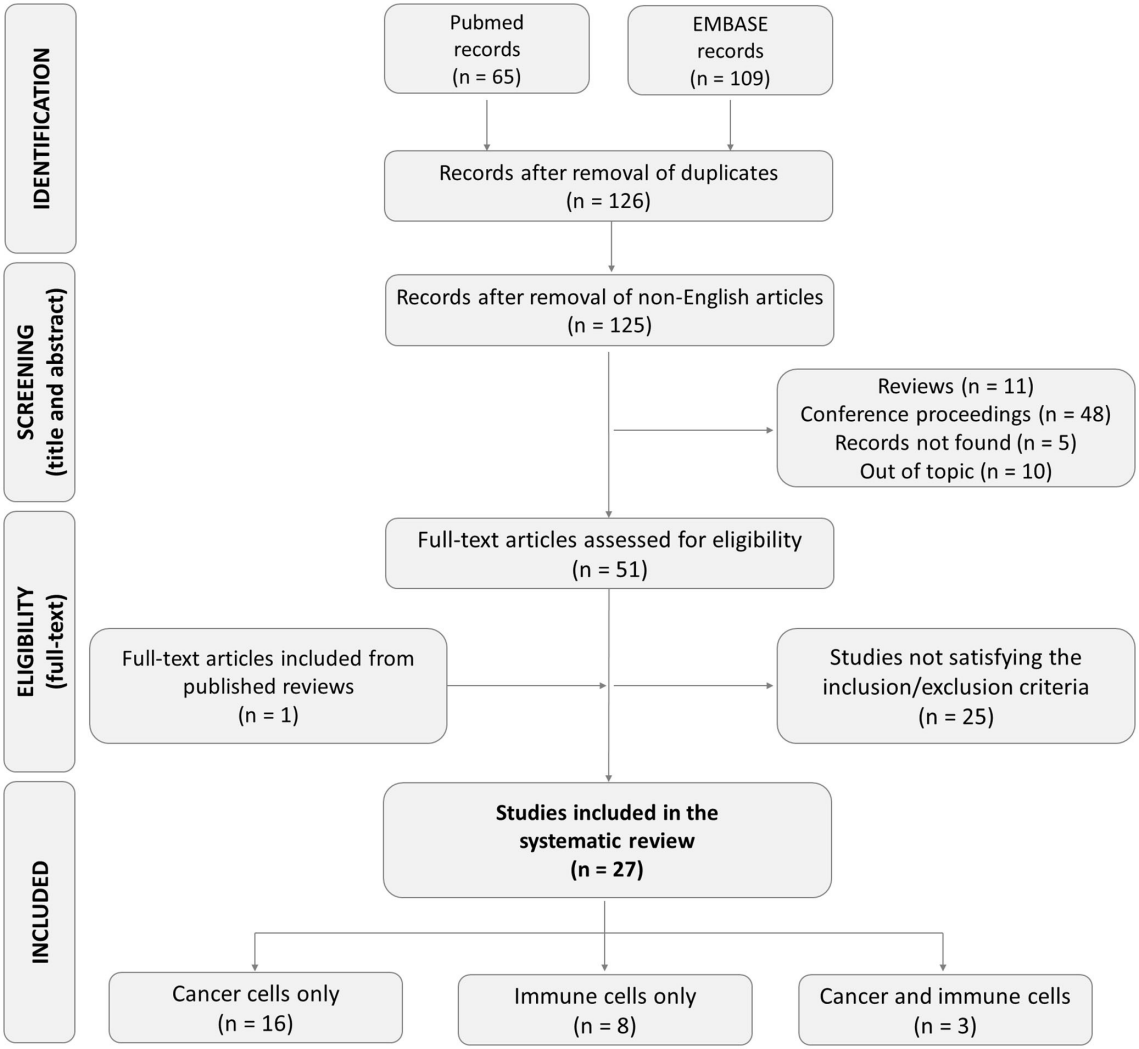
Flow diagram of the study selection process.

Based on the literature search strategy, 174 studies were found (65 in PUBMED and 109 in EMBASE). Among them, 48 articles were excluded because they were doubly reported in the literature search. Of the remaining 126 records, one record was excluded because it was a non-English article. Other articles were excluded for different reasons: 5 records not found, 11 review articles, 48 conference proceedings, and 10 articles not satisfying the inclusion criteria. Of the remaining 51 articles, after reading the full-text, 25 were excluded for the following reasons: 3 studies describing non-microfluidic models, one study using animal cells, 5 methodological articles, 7 articles mentioning extravasation, but describing a model lacking endothelial cells, one study describing only the process of intravasation, 7 studies focusing only on the rolling and/or adhesion step and not analyzing the transendothelial migration phase, one study with unclear methodology. One additional eligible study was retrieved from the bibliography of a review and included. Overall, we finally included 27 studies meeting our eligibility criteria for the subsequent analysis (Figure 1). Among these, 16 studies investigated the extravasation of cancer cells only, 8 investigated the extravasation of immune cells only, and 3 studies investigated cancer and immune cell behavior in the same extravasation models. The studies included are reported and described in Tables 1–3.

**Table 1:** Features of the studies focusing on cancer cell extravasation.

**Extravasating cells**	**Endothelial setting**	**Biophysical factors**	**Biochemical factors**	**Environmental factors**	**Cell features**	**Primary outcome(s)**	**Secondary outcome(s)**	**First author Year**
HepG2 (Hepatocellular carcinoma), HeLa (cervical cancer), MDA-MB-435S (breast cancer)	Endothelial monolayer Dermal HMECs	Flow shear stress (0.03 cm/s) to induce cancer cell deformation	-	Matrigel	Cell with different stiffness subjected to deformation	• Cell deformation = TEM	• Cell deformation = INV	Chaw et al., 2007
ACC-M cell aggregates (salivary gland adenoid cystic carcinoma)	Endothelial monolayer HUVECs	-	CXCL12 (200 ng/mL)	Basement membrane extract	-	• CXCL12 ↑ TEM	• CXCL12 ↑ INV	Zhang et al., 2012
HT-1080 (fibrosarcoma), MDA-MB-231 (breast cancer), MCF-10A (breast epithelial cells)	Self-assembled MVN HUVECs	Flow shear stress (range 0.012–0.48 Pa) to perfuse cancer cells	TNF-α (2, 5, 10 ng/mL)	Fibrin gel	Cells with higher or lower MP	• TNF-α ↑ TEM • ↑ MP ↑ TEM	-	Chen et al., 2013
MDA-MB-231 (breast cancer), MCF-10A (breast epithelial cells)	Endothelial channel HMECs	-	-	Collagen gel	-	-	-	Jeon et al., 2013
MDA-MB-231 (breast cancer)	Endothelialized channel HUVECs	-	CXCL5 (12 nM)	Collagen gel Osteo-cells in EVM	-	• Bone μEnv ↑ TEM • CXCL5 ↑ TEM	• Bone μEnv ↑ INV • CXCL5 ↑ INV	Bersini et al., 2014
PC3, BT-474 (prostate cancer)	Endothelial channel HUVECs	Flow shear stress (2.09 dyne/cm^2^) to perfuse cancer cells	-	Collagen gel Pericytes and astrocytes in EVM	-	• ↑ MP ↑ TEM	-	Tourovskaia et al., 2014
BOKL clone MDA-MB-23 (breast cancer), MCF-10A (breast epithelial cells)	Self-assembled MVN HUVECs	Flow shear stress (0.25 dyn/cm^2^) for endothelial cell pre-conditioning	-	Fibrin gel Osteo-cells in EVM or myoblasts in EVM	-	• Bone μEnv ↑ TEM • Muscle μEnv ↓ TEM • Shear stress ↓ TEM	• Bone μEnv = INV • Shear stress ↑ INV	Jeon et al., 2015
MDA-MB-231 (breast cancer), A-375 MA2 (melanoma)	Self-assembled MVN Endothelial monolayer HUVECs	Flow shear stress (5 dyne/cm^2^) to remove non-adherent cancer cells	-	Fibrin/Collagen gel	-	-	-	Chen et al., 2016
MDA-MB-231 (breast cancer)	Endothelial channel HUVECs	-	CXCL12 (300 ng/mL)	Matrigel	-	• CXCL12 ↑ TEM	-	Roberts et al., 2016
MDA-MB-231 (breast cancer), T24 (bladder cancer), OVCAR-3 (ovarian cancer)	Endothelial channel HUVECs	-	-	Collagen gel Osteo-cells in EVM	Cancer cells with different MP	• Bone μEnv ↑ TEM • ↑ MP ↑ TEM	• Bone μEnv ↑ INV • ↑ MP ↑ INV	Bersini et al., 2018b
MDA-MB-231, LM2-4175 (breast cancer)	Endothelial channel HUVECs	Fluid flow (0.2 μL/s) to perfuse cancer cells	-	Collagen gel	-	-	-	Bertulli et al., 2018
MDA-MB-231, HS578T, MCF7 (breast cancer)	Endothelial channel HUVECs	Flow shear stress (1–6 dyn/cm^2^) to perfuse cancer cells	-	Collagen gel	Cancer cells with or without hyaluronic acid pericellular matrix	• Pericellular matrix ↑ TEM	• Pericellular matrix ↑ INV	Brett et al., 2018
MDA-MB-231, MCF7 (breast cancer), MCF-10A (breast epithelial cells)	Self-assembled MVN HUVECs	-	-	Fibrin gel	Hypoxic and normoxic cancer cells Cancer cells with different MP	• Hypoxic cancer cells ↑ TEM • ↑ MP ↑ TEM	-	Song et al., 2018
PC9, PC9-BrM3 (lung cancer)	Endothelial monolayer Brain HMECs	Fluid flow (0.1 μL/min) for endothelial cell pre-conditioning	-	Collagen and fibronectin Astrocytes	-	• ↑ MP ↑ TEM	-	Liu et al., 2019
MDA-MB-231 (breast cancer)	Endothelial channel HUVECs	Oscillatory flow (1 Pa, 1 Hz) to stimulate osteocytes in EVM	-	Collagen/Matrigel mix Osteocytes in EVM	Osteocytes stimulated or not by oscillatory fluid flow	• Mechanically-stimulated osteocytes ↓ TEM	• Mechanically-stimulated osteocytes ↓ INV	Mei et al., 2019
MDA-MB-231 (breast cancer)	Endothelial channel HUVECs	Flow shear rate* (10, 20 s^−1^) to perfuse cells *Shear stress (0.08, 0.16 dyn/cm^2^)	TNF-α (50 ng/mL)	Matrigel	-	-	• TNF-α ↑ ADH↑ Flow ↓ ADH	Mollica et al., 2019

*When available, primary and secondary outcomes have been summarized. The symbol indicates if the analyzed factor increases (↑) or decreases (↓), or does not affect (=) any extravasation step (ADH, adhesion; TEM, transendothelial migration; INV, invasion). When a single symbol is present, the increase/decrease is defined respect to a control condition. In the case of factor intensity correlating with effect intensity, two symbols are present to indicate the direct or inverse correlation. When the shear rate was indicated in the paper, we calculated the corresponding shear stress value, considering a medium viscosity equal to 0.00082 Pa × s (Raimondi et al., 2002). (HMECs, human microvascular endothelial cells; HUVECs, human umbilical vein endothelial cells; MP, metastatic potential; EVM, extravascular matrix; μEnv, microenvironment)*.

**Table 2:** Features of the studies focusing on the extravasation of immune cells.

**Extravasating Cells**	**Endothelial setting**	**Biophysical Factors**	**Biochemical factors**	**Environmental factors**	**Cell features**	**Primary outcome(s)**	**Secondary outcome(s)**	**References**
Neutrophils	Endothelial channel Dermal HMECs	-	Fmlp (10, 100, 1000 nM) IL8 (1, 10, 100 ng/mL)	Collagen gel with different stiffness	-	• fMLP ↑ TEM • IL8 ↑ TEM • Effect on TEM : fMLP > IL8 • ↑ EVM stiffnes ↓ TEM	• fMLP ↑ INV • IL8 = INV • ↑ EVM stiffness ↓ INV	Han et al., 2012
Neutrophils	Non-self-assembled MVN Porous membrane separating endothelial cells and EVM HUVECs	Shear rate* (<30, 30–60, 60–120, 120–280 s^−1^) to perfuse cells *Shear stress (<0.25, 0.25–0.49, 0.49–0.98, 0.98–1.48 dyn/cm^2^)	TNF-α (10 U/mL) fMLP(1 μM)	n.d.	-	• fMLP ↑ TEM • Shear rate 30–60 s^−1^ ↑ TEM	• Shear rate > 120 sec^−1^ ↓ ADH	Lamberti et al., 2014
Neutrophils	Endothelial monolayer Human endothelial cell line (hy926)	-	fMLP (10, 20, 50 ng/mL) IL-8 (10, 20, 50 ng/mL) LTB4 (10, 20, 50 ng/mL)	Collagen gel	-	• fMLP ↑ TEM • IL8 ↑ TEM • LTB4 ↑ TEM Effect on TEM : fMLP = LTB4 > IL-8	-	Wu et al., 2015
Neutrophils Neutrophils in whole blood	Endothelial channels HUVECs	Flow shear stress (1, 10 dyn/cm^2^) to perfuse cells	TNF-α (10 ng/mL) fMLP (500 nM)	Collagen gel	-	• fMLP ↑ TEM	• TNF-α ↑ ADH • ↑ Shear stress ↓ ADH	Menon et al., 2017
Neutrophils	Endothelial channel iPSC-ECs	-	*P aeruginosa* (bacterial strain)	Collagen gel	-	• Presence of bacteria ↑ TEM	• Presence of bacteria ↑ INV	Hind et al., 2018
Neutrophils Neutrophils in whole blood	Endothelial channel iPSC-ECs	-	fMLP (10 μM) IL8 (11 μM)	Collagen gel	-	• Effect on TEM for purified neutrophils and whole blood : fMLP = IL-8	• Effect on INV for purified neutrophils : fMLP < IL8 • Effect on INV for whole blood : fMLP > IL-8	Ingram et al., 2018
Neutrophils	Endothelial Channel HUVECs	-	IL8 (n.d.)	Collagen gel	Migratory and non-migratory neutrophils	• IL8 ↑ TEM of migratory neutrophils • EVM continuity (no cells in gel) ↑ TEM	-	McMinn et al., 2019
Monocytes	Endothelial monolayer Porous membrane separating endothelial cells and EVM HUVECs	Fluid flow (400 μL/min) for endothelial cell pre-conditioning	MCP-1 (50 ng/mL)	Titanium microbeads in the extravascular chamber	-	• MCP-1 ↑ TEM • Fluid flow ↑ TEM	-	Sharifi et al., 2019

*When available, primary and secondary outcomes have been summarized*.

*The symbols indicate if the analyzed factor increases (↑), decreases (↓), or does not affect (=) any extravasation step (ADH, adhesion; TEM, transendothelial migration; INV, invasion). When a single arrow is present, the increase/decrease is defined respect to a control condition. In the case of factor intensity correlating with effect intensity, two symbols are present to indicate the direct or inverse correlation. When the shear rate was indicated in the paper, we calculated the corresponding shear stress value, considering a medium viscosity equal to 0.0002 Pa × s (Raimondi et al., 2002). (MVN, microvascular network; HMECs, human microvascular endothelial cells; HUVECs, human umbilical vein endothelial cells; iPSC-ECs, induced pluripotent stem cell-derived endothelial cells; EVM, extravascular matrix)*.

**Table 3:** Features of the studies focusing on the interaction between cancer cell and immune cells in the process of extravasation.

**Extravasating Cells**	**Endothelial setting**	**Biophysical Factors**	**Biochemical factors**	**Environmental factors**	**Cell features**	**Primary outcome(s)**	**Secondary outcome(s)**	**References**
A375 and A375-MA2 (melanoma) and MDA-MB-231 (breast cancer)	Self-assembled MVN HUVECs	Flow shear stress (~1 Pa) to perfuse cancer cells	-	Fibrin gel	Unstimulated and LPS-stimulated neutrophils	• Neutrophils and LPS-stimulated neutrophils ↑ TEM of cancer cells	• LPS-stimulated neutrophils ↑ ADH of cancer cells	Chen et al., 2018
MDA-MB-231 (breast cancer) and MDA-MB-435 (melanoma) Monocytes	Self-assembled MVN HUVECs	-	-	Fibrin gel Lung fibroblasts in EVM	Inflammatory and patrolling monocytes	• Inflammatory monocytes ↑ TEM respect to patrolling monocytes • Intravascular monocytes ↓ TEM of cancer cells • Extravascular monocyte-derived • macrophages = TEM of cancer cells	-	Boussommier-Calleja et al., 2019
MDA-MB-231 (breast cancer) Monocytes	Endothelial channel HMECs	-	-	Collagen gel	-	• Extravasating monocytes ↑ TEM of cancer cells • Extravascular monocyte-derived • macrophages ↑ TEM of cancer cells	• Extravascular monocyte-derived macrophages ↑ INV of cancer cells	Kim et al., 2019

*When available, primary and secondary outcomes have been summarized. The symbols indicate if the analyzed factor increases (↑), decreases (↓), or does not affect (=) any extravasation step (ADH, adhesion; TEM, transendothelial migration; INV, invasion). When a single symbol is present, the increase/decrease is defined respect to a control condition. In the case of factor intensity correlating with effect intensity, two symbols are present to indicate the direct or inverse correlation (MVN, microvascular network; HMECs, human microvascular endothelial cells; HUVECs, human umbilical vein endothelial cells; EVM, extravascular matrix)*.

### Quality and Risk of Bias Assessment

**Figure 2:**
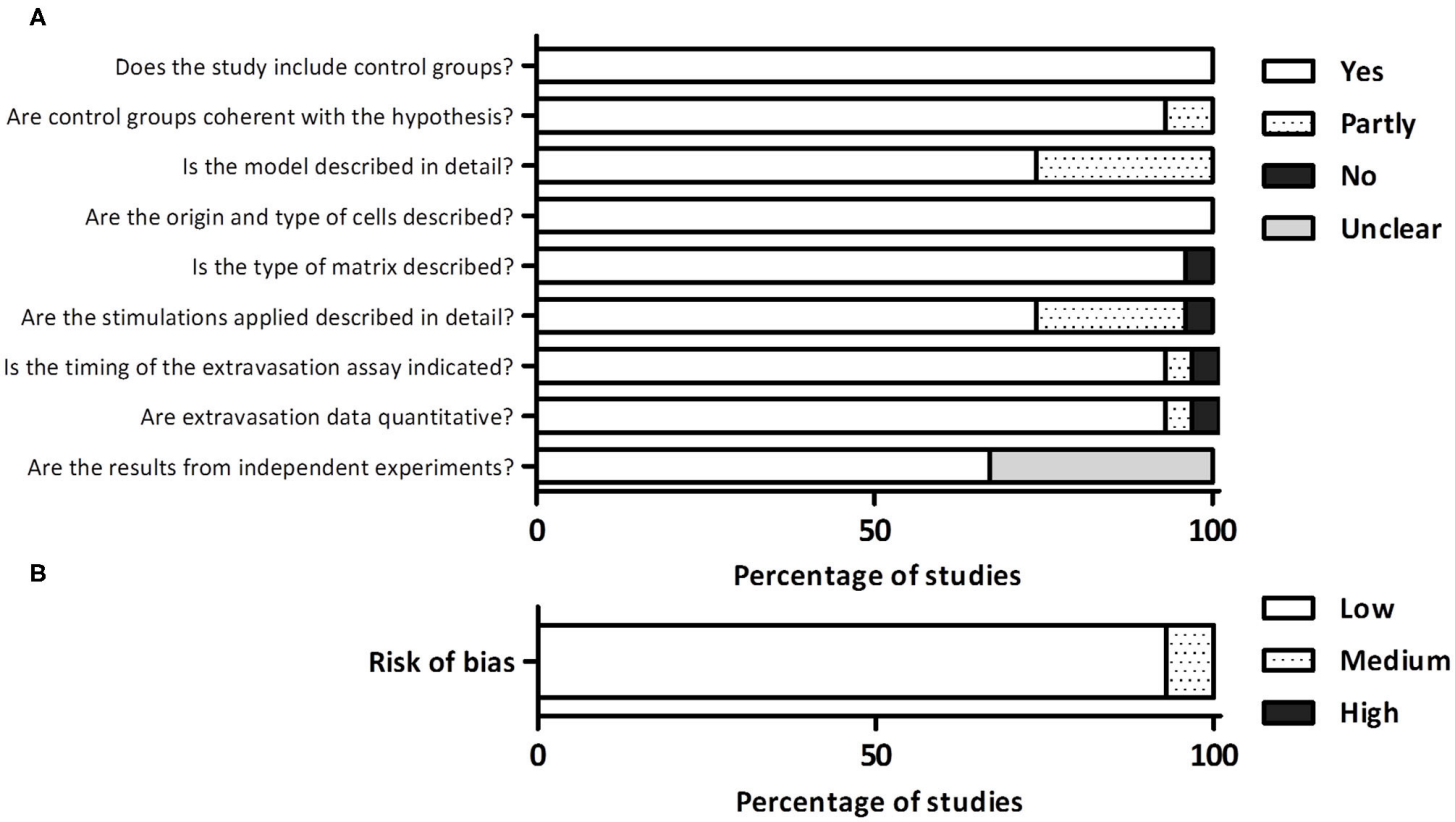
**(A)** Detailed quality assessment evaluating different aspects related to the study design, reporting, and detection phase. **(B)** Overall evaluation of the risk of bias.

The overall quality of the study was high (Figure 2A). For what concerns the study design, all the studies included control groups and in the vast majority of cases, the control groups were highly coherent with the scientific hypothesis. A higher heterogeneity was observed analyzing methodological reporting elements. About 25% of studies (7/27) did not provide a detailed and easily comprehensible description of the microfluidic model, which in some cases impeded the full comprehension of the results. All the studies clearly indicated the origin and type of cells, and the vast majority indicated the type of matrix included in the model. On the other hand, the detailed description of any biochemical and/or biophysical stimulation applied in the system was incomplete or missing in 25% of studies (7/27), leading to the impossibility to fully understand the experimental set-up. Almost all the studies (26/27) described the timing of the extravasation assay. Finally, regarding the detection phase, almost all the studies (26/27) applied a quantitative method to analyze cell extravasation, although 33% of studies (9/27) did not clearly indicate whether the data were obtained from independent experiments. Based on these elements, we classified 93% of studies (25/27) as affected by low risk of bias and 7% (2/27) by moderate risk of bias (Figure 2B).

### Microfluidic Models Investigating Cancer and Immune Cells Extravasation

**Figure 3:**
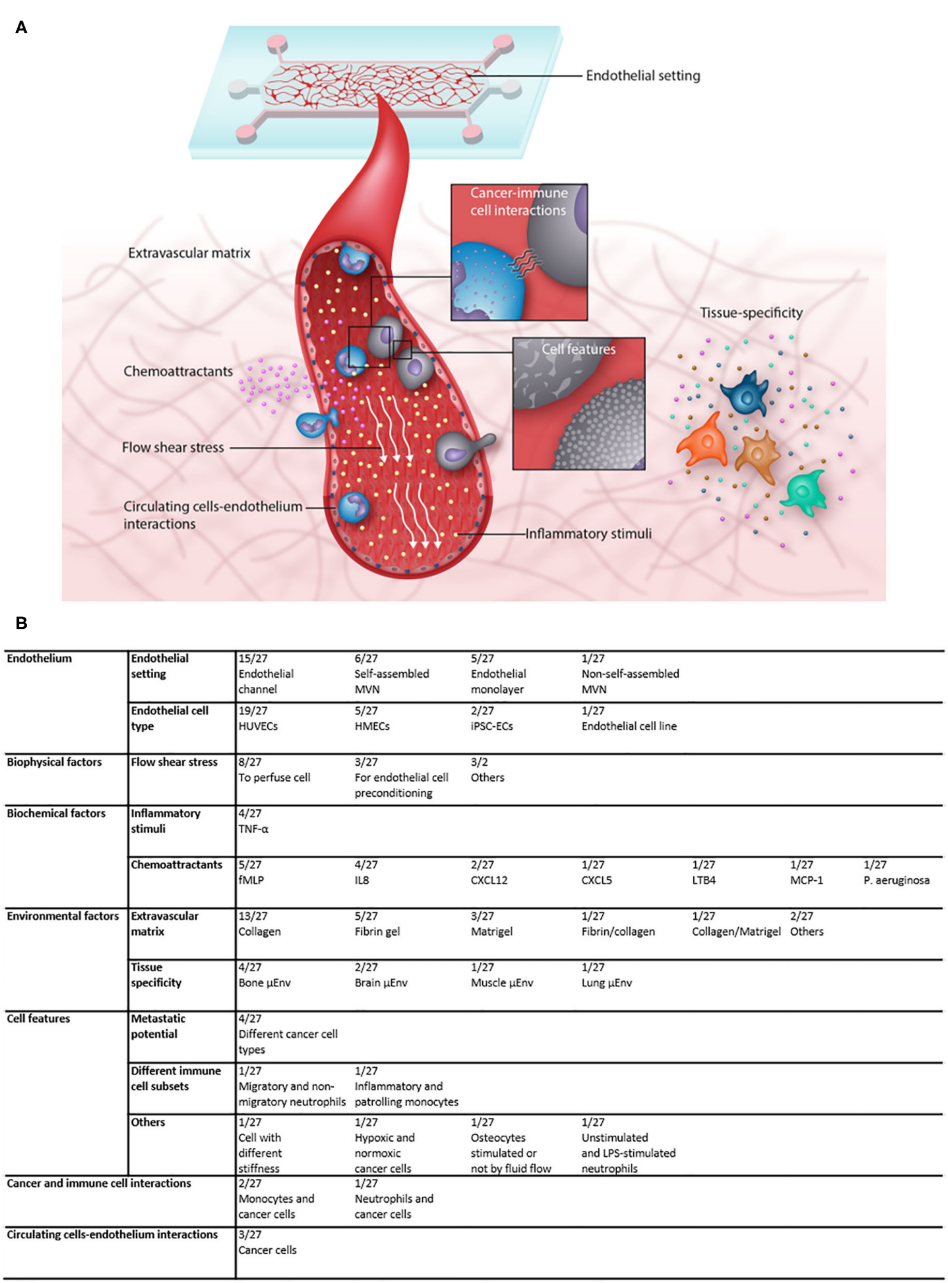
**(A)** Illustration showing the multiple factors that contribute to the complexity of microfluidic extravasation models and the factors that can be investigated in such models. **(B)** Table recapitulating the features of the analyzed articles and the frequency with which each factor was applied and/or analyzed.

Cell extravasation is a multi-step process, subjected to influences deriving from biochemical and biochemical factors and from the microenvironment in which cells extravasate, but also from intrinsic cell properties and the interactions between cells. Different microfluidic models have been designed to consider the specific effects of those factors on the extravasation process and in the following sections we will describe the models applied and the results achieved for each category of factors (Figures 3A,B).

#### Effect of Biophysical Factors

The extravasation process takes place in an environment in which both extravasating cells and endothelial cells are exposed to the frictional force of the blood flow, called shear stress. Shear stress has a crucial role in regulating the interactions between leukocytes and endothelial cells during both physiological and pathological processes. In physiological conditions, leukocyte extravasation mostly occurs in post-capillary venules under shear stress conditions ranging from 1 to 5 dyn/cm^2^ (Bianchi et al., 2013), whereas in some pathological conditions, such as atherosclerosis, leukocytes extravasate through the artery walls where the shear stress is much higher (Schimmel et al., 2017). Shear stress plays also a role in regulating tumor invasion and metastatic spreading. Cancer cells adhere to the endothelium and extravasate mainly within the range of 0.5–15 dyn/cm^2^, shear stress conditions that are reached in capillaries, in veins, and in some arteries (Huang et al., 2018).

To study the effect of the shear stress on the extravasation process, advanced 3D microfluidic models have been proposed due to their compatibility with the application of a controlled shear stimulation, which can mimic the conditions experienced by cells *in vivo* (Zervantonakis et al., 2011). Moreover, microfluidic models can reproduce vessels with specific dimensions and geometrical features (e.g., bifurcations, curvatures, occlusions, etc.) that affect the fluid motion, thus giving the possibility to study the extravasation process in the most varied flow conditions, typical of healthy or pathological states (Wang et al., 2018).

Among the 27 studies included in this review, 13 included a fluid flow applied to the vascular compartment for different purposes: 3 studies used the fluid flow to pre-condition the endothelium before the extravasation assay, 8 to perfuse cells, 1 to induce cell deformation and one to remove non-adherent cells. Of those, only 5 studies analyzed the effect of the flow stimulation compared to a control group, all applied the fluid flow by means of a syringe pump.

To generate the vessels within microfluidic models, two different approaches are mainly used: in the first one, endothelial cells are introduced in a pre-formed microfluidic channel to generate an endothelialized channel, while in the other case endothelial cells embedded in a hydrogel form a 3D microvascular network by self-assembly. In both cases, a 3D endothelial structure with a lumen is obtained mimicking the patency of native vessels. However, great differences exist between these two configurations. If the generation of endothelialized channels with well-defined geometry and dimensions allows controlling the shear stress imposed at the endothelium wall, the second strategy allows a better reproduction of the physiological geometrical structure of native vessels (Wang et al., 2018). To achieve a high control of the flow rate and, therefore, of the shear stress applied at the endothelium wall, the use of syringe pumps is recommended, even though it renders the experimental set-up more complex. A simpler, but less controllable, method exploits the difference in hydrostatic pressure between two reservoirs at the inlet and outlet of the channel, which generates a flow in the channel, defined as gravitational flow. Computational simulations can be carried out to precisely assess the shear stress within the microfluidic channels and correlate the values to the extravasation outcomes.

Shear stress has been shown to influence different steps of the extravasation process of both leukocytes and cancer cells as well as to modify the endothelium properties. Leukocyte extravasation was investigated in dynamic conditions within a synthetic microvascular network, composed by channels mimicking the dimension of post-capillary venules, the main focal sites of leukocyte-endothelium interactions. The microvascular network was obtained through soft-lithography techniques, starting from a digitalized image of the *in vivo* microvascular topology to reproduce a realistic geometry. In this model, computational fluid dynamic analysis allowed defining the shear stress values of specific regions to correlate the shear stress with either cell adhesion or transendothelial migration outcomes. In particular, the correlation between adherent and extravasated cells with shear stress values showed that neutrophil adhesion was reduced in regions where the shear rate was higher than 120 s^−1^ (corresponding to an estimated shear stress of 0.98 dyn/cm^2^) and that transendothelial migration mainly occurred in regions characterized by a shear rate between 30 and 60 s^−1^ (corresponding to an estimated shear stress of 0.25–0.49 dyn/cm^2^), as compared to regions with higher values. Rolling velocities measured from videos of neutrophils flowing in the microchannels, showed values closely mimicking the *in vivo* situation, corroborating the findings of this study (Lamberti et al., 2014). A shear stress lower than 0.1 dyn/cm^2^ was also reported to enhance the positive effect of the Monocyte Chemoattractant Protein-1 (MCP-1) on monocyte transendothelial migration (Sharifi et al., 2019), suggesting that different types of leukocytes have a similar behavior in response to low shear stress conditions. Similar evidences emerged from a study in which a microfluidic model has been used to reproduce the stenotic occlusion typical of atherosclerosis and investigate the effect on neutrophil adhesion of flow and inflammation within the obstructed channel. Neutrophil adhesion along the channel was minimal at 1 dyn/cm^2^ in healthy conditions, while it was enhanced when an inflammatory stimulus was present. On the other hand, neutrophil adhesion at higher shear stress values (10 dyn/cm^2^, value in the physiological shear stress range found in the arterioles, where atherosclerosis is favored), remained low along the channel even in the presence of an inflamed endothelium, indicating that the activation of the endothelium was not sufficient to counterbalance the effect of high shear stress. Differently, when neutrophils encountered obstacles in their trajectory, such as an obstruction in the microchannel mimicking the stenotic condition, they could adhere to the endothelium despite high shear stress levels (Menon et al., 2017).

Not surprisingly, shear stress has also a major role in cancer cell extravasation and consequently in the formation of tumor metastasis. Low shear stress (0.25 dyn/cm^2^) was shown to reduce breast cancer cell transendothelial migration compared to the static condition, whereas it increased the migration distance of cancer cells in the extracellular environment possibly due to the generation of an interstitial flow within the extravascular environment (Jeon et al., 2015). Shear stress was also shown to negatively correlate with the adhesion of breast cancer cells to the endothelium, since a shear stress of 0.16 dyn/cm^2^ within an endothelialized channel reduced cancer cell adhesion compared to a half-shear stress value (0.08 dyn/cm^2^). This trend was present also when an inflammatory stimulus was added, once again showing the prevalent effect of shear stress over inflammation, although a higher number of cells adhered to the endothelium compared to a healthy condition (Mollica et al., 2019).

Besides the direct influence on the extravasating cells, shear stress can also have an indirect effect, through the modification of endothelial properties. A shear stress of 0.25 dyn/cm^2^ applied in a microvascular network decreased the endothelial permeability compared to static conditions, possibly due to the tightening of endothelial cell junctions (Jeon et al., 2015). In line with this result, 10-fold lower shear stress values applied on an endothelial monolayer were sufficient to induce the expression of VE-cadherin and ZO-1, both involved in the formation of endothelial cell junctions (Liu et al., 2019). However, a minimal shear stress threshold to induce modifications in endothelial cell behavior seems to exist, since VE-cadherin and ICAM-1, an adhesion molecule involved in leukocyte-endothelial cells interactions, were not upregulated by a 100-fold lower value of shear stress (Sharifi et al., 2019). Altogether, these results suggest that shear stress is important for the formation of an intact endothelial barrier mediating the first step of the extravasation process, even though the values of shear stress tested *in vitro* are far below the physiological values.

Although the shear stress stimulation is mostly used to mimic the flow conditions to which endothelial cells are exposed *in vivo*, it can also be used to mechanically stimulate the cells of the extracellular environment to investigate its effect on the process of cell extravasation. To this purpose, an oscillatory fluid flow (1 Pa, 1 Hz) was applied to stimulate osteocytes mimicking the interstitial shear stress experienced by bone cells *in vivo*. When bone cells were stimulated, the extravasation and migration distance of breast cancer cells decreased, highlighting how biophysical stimulation, modifying the behavior of the extravascular environment, can play a role in the extravasation of cancer cells (Mei et al., 2019). Beside shear stress, other biophysical stimuli can affect the extravascular environment and, subsequently, cell extravasation. For instance, microfluidic models applying mechanical stimuli, such as compression (Occhetta et al., 2019) or stretch/strain (Gaio et al., 2016), have been recently developed showing important effects on the stimulated tissues. Similar technological solutions could be also implemented in models designed to investigate cell extravasation to correlate the behavior of extravasating cells with the reaction of the extravascular environment to biophysical stimuli. Furthermore, recent advances in microengineering have allowed the generation of more physiologically relevant microenvironments in which more than one type of biophysical stimuli can be applied (Kaarj and Yoon, 2019). However, despite these recent advances and the potential of microfluidic models to implement a variety of biophysical stimuli, among the 27 articles analyzed in this review, shear stress was the only biophysical stimulus that was investigated in relation to the cell extravasation process and, for this reason, the only one described here.

Summarizing, higher shear stress values decreased the extravasation of both immune and cancer cells, reducing their adhesion to the endothelium and increasing the tightness of the endothelial barrier. The ability of microfluidic systems to apply shear stress in vascularized channels allowed evaluating differences in endothelial permeability and in cell extravasation, which are hardly detectable with other models. Furthermore, the possibility to implement channels with a desired geometry highlighted that shear stress cannot be considered as a single element affecting the extravasation process, but it should be analyzed in combination with the geometrical features of the vascular environment because these two elements act together in determining cell trajectories and the probability of the circulating cells to adhere to the endothelium and, hence, transmigrate.

#### Effect of Biochemical Factors

Among different biochemical stimuli present in the microenvironment, those related to inflammation and chemotaxis play a key role in the extravasation of immune and cancer cells. Inflammation is a body response activated by toxic stimuli and pathological conditions, such as infections or tissue injuries (Medzhitov, 2008), but it is also a hallmark of cancer, involved in the development and progression of malignancies (Diakos et al., 2014). Inflammation influences cell extravasation mainly through a modulation of endothelial properties, decreasing the tightness of endothelial junction (Mollica et al., 2019) and leading to a higher permeability of the endothelial barrier (Chen et al., 2013; Menon et al., 2017; Mollica et al., 2019). Chemotaxis is fundamental for the homeostatic trafficking of immune cells and for the enrollment of leukocytes to infection and inflammation sites (Luster, 2001). Chemotaxis is also involved in each step of cancer spread, leading to metastasis formation (Roussos et al., 2011). Chemoattractants and their receptors act as mediators in the chemotaxis of cancer cells, stromal cells, and cancer-associated inflammatory cells. When the regulation of these mediators is unbalanced, spreading, and progression of cancer is favored, determining a dramatically more efficient cancer dissemination (Roussos et al., 2011).

Among the 27 selected articles, 13 studies investigated the effect of biochemical stimulations on the extravasation process. Among these, 4 studies applied an inflammatory stimulus and 10 tested the effect of chemoattractants.

Microfluidic models are highly suitable for investigating the effects of biochemical stimuli on extravasation because they allow introducing external factors in the system to model specific *in vivo* conditions. This strategy consents to achieve the spatial and temporal control of biochemical gradients at relevant dimensions and time scales, which could not be obtained in traditional 2D cultures. The use of microfluidic models has allowed recapitulating gradients of hormones, growth factors, cytokines, and other biomolecules that play a fundamental role in processes such as angiogenesis, tumorigenesis, and cells migration, leading to an improvement in understanding of cells motility due to chemical gradients (Young and Beebe, 2010).

With regards to the investigation of the role of inflammation in extravasation, microfluidic models consent to generate gradients of inflammatory factors, usually TNF-α. These factors can be directly diluted into the culture medium (Chen et al., 2013; Menon et al., 2017) or incorporated into a gel (Mollica et al., 2019). The latter approach better mimics the physiological origin of the inflammatory stimuli, which usually takes place deep in the tissue, and eventually reaches the endothelial barrier. When supplemented directly in the medium, TNF-α is usually added in a concentration of 10 ng/mL, whilst its addition in the gel needs a higher concentration to reach a biological effect (Mollica et al., 2019). It is fundamental to apply the correct dose of inflammatory factors, considering that they can be cytotoxic when used at high concentrations, both on extravasating cells and on endothelial cells. As for the biological effect, also the cytotoxic effect depends on the mode of application and on the timing. A concentration of 10 ng/mL directly imposed on endothelial cells was reported to be toxic, leading to cell death and ruptures in a self-assembled microvasculature (Chen et al., 2013), while no toxic effect occurred at higher concentration of TNF-α (50 ng/mL) when the molecule was included in a gel (Mollica et al., 2019).

Exploiting microfluidic models, it has been demonstrated how TNF-α modulates endothelial intercellular adhesion and increases vascular permeability, allowing to better understand the influence of inflammatory stimuli on cell extravasation. These effects can be directly visualized by perfusing fluorescent dextran or μm-sized fluorescent polystyrene beads into the endothelial channel (Chen et al., 2013; Menon et al., 2017; Mollica et al., 2019). The increase of the endothelial barrier permeability indicates a loosening of cell junctions and correlates with an increase in cancer cells transendothelial migration (Chen et al., 2013). Another effect of inflammation that has been observed in microfluidic models is the higher expression of specific molecules by endothelial cells, such as adhesion receptors and integrins, which promotes cancer-endothelial cell adhesion and contributes to increase the number of extravasated cancer cells (Mollica et al., 2019). Inflammatory stimuli have been also applied to promote the interaction between neutrophils and endothelium, which are essential to reproduce the pathological inflammatory state typical of cardiovascular diseases (Menon et al., 2017).

Beyond inflammatory factors, microfluidic models permit to create gradients of chemoattractants, by incorporating them directly into the extravascular matrix (Han et al., 2012; Menon et al., 2017; Sharifi et al., 2019) or by diluting them into the medium placed in a separate compartment or channel of the microfluidic device (Lamberti et al., 2014; Wu et al., 2015; Roberts et al., 2016; Ingram et al., 2018). The profile and the stability of the chemical gradient can be simulated with a FEM (finite element method) software (Han et al., 2012; Wu et al., 2015), and the results can be easily compared with empirical tests conducted using a fluorescent tracer with a molecular weight comparable to that of the chemoattractant (Wu et al., 2015). As an alternative, chemoattractant factors imposed through the gel can be quantified in the medium collected over time to obtain a release profile (Sharifi et al., 2019).

Chemoattractants with a well-defined effect have been exploited in microfluidic models to validate their own ability to reproduce a specific pathologic microenvironment. In this scenario fMLP (formyl-methionyl-leucyl-phenylalanine) has been used to validate a new microfluidic system to study neutrophil motility and extravasation (Hind et al., 2018). MCP-1 (Monocyte Chemoattractant Protein-1) was used to validate a Foreign Body Response-on-a-Chip platform designed to study monocyte extravasation during implant-induced inflammation (Sharifi et al., 2019). Concerning cancer extravasation models, a gradient of SDF-1α (Stromal Derived Factor-1α) was applied to induce the extravasation of breast cancer cells and validate a platform for the study of single cell extravasation (Roberts et al., 2016).

Through the design of the microfluidic device, chemoattractant factors can also be studied in competition with each other to establish a chemoattractant hierarchy. The superior chemoattractant effect of fMLP as compared to IL8 (Interleukin-8) was proven analyzing neutrophils movement during the initial transendothelial migration phase and in the subsequent migration into the extravascular matrix (Han et al., 2012) in the presence of both molecules. Using another model, where neutrophils were simultaneously subjected to the stimuli of two diverse factors coming from two opposite ends of the migration channel, a similar chemoattractant hierarchy was proven. LTB4 and fMLP showed a similar ability in inducing neutrophil transendothelial migration, which was higher compared to that of IL8, in line with findings showing a prevalent effect for LTB4- and fMLP-activated signaling pathways (Wu et al., 2015).

Agents targeting chemotaxis have been tested in microfluidic models with the aim to the develop new therapeutic strategies. Human monoclonal anti-FPR1 antibody and Wortmannin (a fungal metabolite), were shown to be effective in impeding neutrophil migration to fMLP (Han et al., 2012; Lamberti et al., 2014). A similar approach was applied to hinder the migration of cancer cells, using a CXCR4 (CXCL12 receptor) antagonist, which blocked the transendothelial migration of cancer aggregates (Zhang et al., 2012). Finally, cancer cell extravasation was blocked through the use of an antibody directed against a cancer cell surface receptor (CXCR2) specific for a chemokine, CXCL5, secreted by osteoblastic cells (Bersini et al., 2014).

To summarize, in the analyzed articles the effect of an induced inflammatory state was mainly studied in relation to its effect on endothelium activation and permeability, while the effect of chemoattractants was tested with different purposes depending on the scientific hypothesis of the study. In models investigating immune cells, chemoattractants were mainly applied to validate new microfluidic models or test different chemoattractant factors in competition. In cancer cell extravasation models, the studies were more focused in identifying strategies to block the effect of chemoattractants on cancer cells, with the final goal of finding a strategy to impede the metastatization process. The possibility to measure endothelial permeability in the context of a 3D microenvironment allowed correlating the modification of endothelial junctions induced by inflammatory factors with an increased cell extravasation. Furthermore, the possibility to generate controllable and measurable gradients of chemotactic factors empowered the identification of molecules and signaling pathways involved in the cell extravasation process, that can be pharmacologically inhibited or enhanced depending on the desired outcome. That leads to the exploitation of these models as platforms for the screening of new therapeutic agents.

#### Effect of Environmental Factors

The reproduction of some characteristics and properties of tissue-specific microenvironments in particular the 3D structure, in a microfluidic model is obtained through the use of hydrogels to form an extravascular matrix (EVM). *In vivo*, the EVM is a dynamic and intricate structure composed by hundreds of diverse proteins, including matrix proteins, growth factors, cytokines, and several bioactive products deriving from matrix degradation and impacting cellular differentiation, proliferation, and migration (Boyd and Thomas, 2017). In microfluidic models, the EVM is not so complex, but still provides cells with a 3D environment which facilitates the maintenance of cell function and is enriched over time by cell-produced molecules. The introduction of a 3D EVM in microfluidic devices for the study of extravasation represents a step forward compared to standard models, such as transwell models, due to a better reproduction of a functional endothelial barrier and the possibility to decouple the results from the gravitational effect. The EVM can also embed tissue-specific cells, allowing to reproduce even better a tissue-specific microenvironment in microfluidic models.

Among the 27 articles included in this review, 8 articles correlated the cellular extravasation process to the features of the extravascular matrix and 7 introduced tissue-specific cells to study the extravasation of cancer cells. Among these, 5 studies dissected the contribution of tissue-specific cells to the extravasation process compared to a control condition.

Collagen and fibrin gel, alone or in combination, are the hydrogels mainly used to reproduce the EVM. The use of collagen and fibrin allows modulating the mechanical features of EVM, since it is possible to vary collagen stiffness by adjusting the pH before the polymerization (Han et al., 2012), whilst fibrin stiffness can be easily tuned by modulating the concentrations of fibrinogen and thrombin as well as the polymerization conditions (Ingram et al., 2018). Alternatively, Matrigel, which is a widely used substrate for endothelial cell culture and angiogenesis assays, has been also employed (Chaw et al., 2007; Roberts et al., 2016).

The choice of EVM is crucial, considering its role in the development of endothelial vessels. Vessels formed in fibrin gel show morphological elongation, characterized by tight cell junctions and vessel maturity, while vessels formed in collagen matrix lack morphological elongation, showing a leaky vessels phenotype and higher permeability, as demonstrated by FITC-dextran perfusion experiments (Ingram et al., 2018). These differences are particularly relevant considering the close correlation between the tightness of the endothelium and cell extravasation.

Beyond the effects on the endothelium, stiffness and porosity of the matrix can directly affect cancer and immune cell extravasation. Firstly, these matrix properties affect the passive diffusion of biochemical gradients imposed across the EVM. Additionally, EVM stiffness can directly influence cancer cell motility since cells tend to move toward more rigid ECM, with a process called durotaxis (Roberts et al., 2016). On the contrary, matrix stiffness can represent an obstacle for cell migration, as shown in the case of neutrophils whose migration was decreased in correspondence of higher stiffness of collagen matrix (Han et al., 2012).

The introduction of tissue-specific cells allows reproducing different tissue-specific microenvironments. Bone tissue represents one of the most investigated extravasation site, being acknowledged as a fertile metastatic target for various tumors, such as breast, prostate, thyroid, lung, bladder, renal carcinoma, and melanoma (Arrigoni et al., 2016, 2017; Macedo et al., 2017). The first reported approach aimed at reproducing bone tissue in microfluidic models was based on the inclusion of osteo-differentiated bone marrow-derived mesenchymal stems cells in a hydrogel matrix. Using this strategy, the extravasation and migration of breast cancer cells was shown to be higher in a bone microenvironment compared to empty EVM (i.e., without any cell), used as a control to prove that tissue specific cells do play a role in the process (Bersini et al., 2014). Additionally, bone- and muscle-mimicking microenvironments were compared, demonstrating the preferential extravasation of breast cancer cells toward the bone microenvironment (Jeon et al., 2015). These results are in accordance with the clinical data that indicate bone and muscle as a preferential and non-preferential metastatization site for breast cancer, respectively, proving that the model recapitulates the *in vivo* situation. The use of these muscle-specific models allowed also identifying the adenosine secreted by myoblasts as a protective agent against metastatization, leading to a better insight into the mutual interactions between microenvironment and extravasating cells (Jeon et al., 2015). The comparison between these two tissues also showed that the network developed into the muscle-mimicking microenvironment is characterized by higher permeability compared to the bone-like microenvironment, although the cancer extravasation rate was higher in the latter. This result decouples the direct correlation between the endothelial permeability and the extravasation rate, proving that this process is affected by multiple factors, among which the microenvironmental conditioning and the presence of cell-secreted molecules play a critical role. *In vivo*, tissues are exposed also to specific mechanical stimuli, which are particularly important in the case of bone tissue. To study their influence on metastatic cell extravasation, bone cells cultured on a layer of collagen I in the EVM compartment were exposed to an oscillatory flow, to generate a mechanically-stimulated bone microenvironment that was compared to the unstimulated conditions. A decrease in the extravasation rate and migration distance of breast cancer cells was shown in the case of mechanical stimulation of bone cells, showing a protective effect of mechanical loading against the formation of bone metastasis (Mei et al., 2019).

The brain environment has been also object of study since brain metastases are among the most lethal events in cancer progression. In this context, to mimic *in vitro* the brain parenchyma toward which cancer cells extravasate, astrocytes were introduced in the EVM. The presence of brain specific cells combined with flow stimulation increased the tightness of brain-specific microvessels forming the blood-brain barrier by decreasing the endothelial permeability if compared to conditions without astrocytes and shear stimulus. These results indicate that astrocytes modify the endothelium properties and that this might change the cancer cell extravasation outcomes, approaching a condition more similar to the *in vivo* situation (Liu et al., 2019).

While tissue-specificity represents a key factor driving cancer cells extravasation, there is no evidence that it significantly influences the extravasation of leukocytes. Leukocyte extravasation has been generally considered as uniform between different tissues. However, in some organs the mechanisms guiding leukocyte extravasation can differ from the classical leukocyte adhesion cascade due to a peculiar structure of the endothelium, which can be fenestrated or discontinuous (e.g., in the bone marrow), and for the proteins involved in the different steps of leukocyte extravasation process (Maas et al., 2018). Among the analyzed studies, none investigated the influence of tissue-specific microenvironments on immune cell extravasation, probably due also to the difficulty in finely reproducing *in vitro* an endothelium with tissue-specific features in terms of endothelial cell organization and tightness.

In this context, beyond the presence of tissue-specific cells in the model, the presence of a tissue-specific endothelium would allow the study of cancer cell and leukocyte extravasation with a higher reliability, since the phenotypic traits of the endothelium strongly depends on its anatomical origin (Marcu et al., 2018). One of the studies analyzed in this review introduced a brain-specific endothelium mimicking the blood-brain barrier (Liu et al., 2019). However, the results on extravasation were not compared with those obtained with a non-specific endothelium, which did not allow a direct confirmation of the effects of a tissue-specific endothelium on extravasation. Among the 27 articles included in this review, 19 reproduced the endothelium by using HUVECs, regardless of the tissue under investigation. Indeed, HUVECs represent the most common source of primary endothelial cells used in *in vitro* models (Kocherova et al., 2019) due to the wide availability and easier culture protocols. On the contrary, tissue-specific endothelial cells are scarcely available commercially and difficult to isolate from human tissues, which hampers their easy translation into *in vitro* settings. To overcome these major limitations, a possible alternative to the isolation of tissue-specific endothelial cells is the induction of a tissue-specific phenotype in HUVECs, by co-culturing them with tissue-specific cells, which has been shown to promote the acquisition of endothelial phenotypic tissue-specificity (Visone et al., 2016; Bersini et al., 2018a).

Summarizing, the presence of vascular channels or vascular networks associated to a tissue-specific environment in microfluidic models allowed evidencing the effect of EVM and tissue-specific cells on the properties of endothelium and on cell extravasation. The possibility to tailor the microenvironment (e.g., modifying matrix mechanical properties or including cells secreting tissue-specific molecules) allowed the study of the crosstalk between circulating and tissue-specific cells, thanks to the possibility of testing multiple combinations of cells in a standardized environment where circulating and tissue-specific cells are the only variables. Although this approach neglects some fundamental factors that play a role *in vivo*, such as the degree of vascularization, tissue-specific matrix composition and endothelium structure, it allows shading light on protective factors secreted by tissue-specific cells as well as on factors determining the pro-metastatic behavior of some tissues, which could find an application as preventive therapy against cancer metastatization.

#### Effect of Cell Features and Interactions Between Cancer and Immune Cells

The fate of a circulating cell from blood vessels to extravascular tissue-specific environments is not determined only by biochemical, biophysical, and environmental features, but also by own cell features that can determine its probability to extravasate. The intrinsic ability of a cancer cell to extravasate is related to its metastatic potential, defined as the ability to invade specific secondary organs. Similarly, leukocytes have different ability to extravasate during inflammation based on their phenotype and function. Among single cell features determining the ability to extravasate, cell deformability has been particularly studied, since it influences different steps of the metastatic cascade in which cells leave the primary site of tumor, circulate in the bloodstream, and extravasate (Swaminathan et al., 2011). Indeed, cancer cells deform to pass through endothelial cell junctions during both the intravasation and the extravasation processes and they need also to deform to avoid being stuck in small capillary vessels (Hu et al., 2018). Beside the properties of single circulating cells, the interplay between cancer cells and leukocytes is increasingly being investigated to determine its influence on cancer extravasation and metastasis formation. Leukocytes have been shown to be actively involved in tumor progression and metastasis formation with conflicting results. In some cases leukocytes have been shown to promote the extravasation of cancer cells (Liang et al., 2010), whilst in other cases they have been shown to hamper cancer dissemination by provoking tumor death, thus representing interesting candidates for the development of immunotherapies (Lanca and Silva-Santos, 2012).

Among the 27 studies included in this review, 4 studies investigated the effects of the cancer cell properties on extravasation, 2 papers studied the influence of leukocyte subpopulation (1 for monocytes and 1 for neutrophils), and 3 investigated the interactions between leukocytes and cancer cells.

To study the effect of circulating cells properties and of their interactions on the extravasation process, microfluidic models are key tools, since they permit to visualize cell deformation and interactions within an *in vivo*-like environment through high resolution imaging techniques. Furthermore, microfluidic models can well reproduce the organization of the intravascular and extravascular spaces if compared to standard 2D culture systems, allowing to monitor if leukocytes affect the metastasis formation when they are inside the vessels and/or when they are already infiltrated in the tissues. As mentioned before, the microfluidic models that can be used to investigate these processes can be classified in two different types, being either based on an endothelialized channel in which an endothelial monolayer divides the EVM from the intravascular space or through gel-embedded endothelial cells self-assembling in a 3D microvascular network surrounded by the EVM. This latter system, beyond providing a more physiological-like structure of the vessels, also allows a better real-time monitoring of cell interactions (e.g., clusters) within the vessel or with the endothelium.

To study the influence on extravasation of cell deformation in small vessels, microfluidic models including 10 μm micro-gaps mimicking blood capillaries were generated. Different types of cancer cells injected through these capillaries-like structures under flow showed a different mechanical deformation. Higher cell stiffness was correlated with higher cell viability after deformation, but it did not result in a different number of extravasated cells or migration distance (Chaw et al., 2007). The possibility to monitor in real-time cell transendothelial migration within a microvascular network allowed studying how cancer cells deform while crossing the endothelial barrier. Additionally, differences were observed in the extravasation of single circulating cells compared to cell clusters and of adherent cells compared to mechanically trapped cells (Chen et al., 2013).

Besides investigating the effects of cell deformation, microfluidic models were exploited to distinguish the extravasation ability of cancer cells with a different degree of malignancy (Chen et al., 2013) as well as of cancer cells originating from different primary tumors (Tourovskaia et al., 2014). For instance, the presence of hyaluronan in the pericellular matrix of breast cancer cells (Brett et al., 2018) or the expression of specific proteins implicated in brain cancer development (Liu et al., 2019) were studied in relation to the metastatic potential of cancer cells, demonstrating that microfluidic extravasation models are able to capture differences in cell malignancy and allow identifying some of the elements responsible of a high metastatic potential. Breast cancer cells with different metastatic potential were also pre-conditioned in hypoxic conditions before injection in a microfluidic device, demonstrating that hypoxic cells have a higher extravasation rate compared to normoxic cells (Song et al., 2018). The primary tumor from which metastatic cells originate can also heavily influence their extravasation potential in relation to tissue-specific metastatization sites. Cells from breast, bladder and ovarian cancer in a microfluidic model displayed a different affinity toward a bone-mimicking microenvironment, with the bladder cancer cells showing the highest extravasation rate and migration distance and the ovarian cancer cells the lowest (Bersini et al., 2018b). These results are in line with clinical evidence demonstrating that different types of cancer preferentially invade different secondary organs.

Similar to cancer cells, leukocytes showed differences in the extravasation potential between different leukocyte subsets. Exploiting a microfluidic system allowing the physical separation of the extravascular and intravascular environment, a separate analysis of extravasated and non-extravasated neutrophils allowed studying the transcriptional profile of migratory and non-migratory neutrophil subsets (McMinn et al., 2019). Another model was applied to investigate the extravasation ability of different monocyte subsets, demonstrating that inflammatory monocytes have a superior TEM rate than patrolling monocytes (Boussommier-Calleja et al., 2019).

During extravasation, cancer cells interact with the endothelium and modify its microarchitecture. In this context, breast cancer cells characterized by invasive phenotype were shown to directly modify the function of the endothelium by increasing endothelial permeability compared to a non-tumorigenic cell line (Jeon et al., 2013). The negative influence of cancer cells in the maintenance of an intact endothelium was observed through fluorescence microscopy imaging. Immunofluorescence analysis allowed to obtain information about the endothelium integrity through the visualization of two tight junction proteins, ZO-1 and VE-cadherin, expressed by endothelial cells, both showing a decreased expression during cancer cell extravasation (Liu et al., 2019). The mechanisms that leads to endothelium disruption by cancer cells have not yet been clarified and can either depend on a local damage of the endothelium by cell contact or on the secretion of factors affecting the endothelial cell junctions. High resolution imaging showed the formation of gaps in the endothelial barrier during the physical passage of cancer cells, followed by intact endothelial cell-cell junctions immediately after the extravasation of cancer cells. This data reveals that the damage of the endothelial barrier is not irreversible, supporting the hypothesis that cancer cells might regulate the endothelial permeability through the secretion of biochemical factors (Chen et al., 2013).

Regarding the interactions between cancer cells and monocytes, both circulating monocytes and monocyte-derived macrophages resident in tissue EVM have been studied within microfluidic models. Circulating monocytes perfused together with breast cancer cells in a microvascular network were shown to decrease cancer cell extravasation rate. Since monocytes did not show prolonged contact with cancer cells in the microvessels, this effect probably depended on paracrine signaling rather than cell-cell contact. On the other hand, monocyte-derived macrophages present in the EVM did not affect cancer cell extravasation, suggesting a role of monocytes in cancer extravasation when they are inside the vessels but not when they are in the tissue (Boussommier-Calleja et al., 2019). Partially in contrast with this last result, human monocytes extravasated to the EVM were shown to promote breast cancer cell extravasation as a result of MMP9 secretion, which caused the disruption of the endothelial barrier, and of the decreased expression of two tight junction proteins, ZO-1 and Occludin. The same study showed that monocyte-derived macrophages generated microtracks in the EVM, thus facilitating cancer cell migration and matrix invasion (Kim et al., 2019). The role of circulating leukocytes in cancer can be also influenced by the presence of inflammatory conditions. Indeed, inflamed neutrophils injected in a microvascular network model aggregated with circulating melanoma cells, facilitating the adhesion of cancer cells to endothelial cells. The confinement of these aggregates to the endothelium was favored by the secretion of IL8 from neutrophils and correlated with a higher extravasation of cancer cells (Chen et al., 2018).

Although the features of cancer and immune cells can be investigated also with standard *in vitro* and *in vivo* models, high-resolution real-time analyses that can be performed in microfluidic models allowed elucidating how certain cell features can impact on the ability of the cell to cross the endothelial barrier and evidencing the mechanisms which are activated during this process. Furthermore, microfluidic models allow studying how cancer and immune cells can interact with each other in the intravascular and extravascular environment, producing an effect on cancer cell extravasation, due to their ability to dissect the effects of cellular interactions during each specific extravasation step.

## Summary

Cell extravasation is a highly regulated process through which cells leave the bloodstream, cross the endothelium and finally migrate into the tissues. As evidenced from the literature, several studies investigated this process for immune or cancer cells exploiting microfluidic devices, which were analyzed and compared in this systematic review. As mentioned above, the overall quality of studies describing the application of microfluidic extravasation models is high. However, improvements could be pursued, mostly from a reporting aspect, in future studies. The understanding of microfluidic models design, as well as the localization of the different cell/matrix components in the systems is not always easy. The use of scientific illustrations, providing a graphical scheme of the model and highlighting how the different elements of the model mimic the *in vivo* situation, would significantly facilitate the comprehension of the studies. Additionally, we encourage authors applying biophysical and biochemical factors in their microfluidic models to describe in detail the methodology. For biochemical factors, it is essential to describe the concentration of the factor and the timing of application. Also, if a biochemical gradient is present, the way it is generated and maintained should be clearly indicated and the gradient should be characterized by computational modeling and/or experiments with fluorescent tracers over time. For what concerns biophysical factors, we found that flow shear stress is the factor most often applied. In this case, we encourage authors to clearly state the purpose and the timing of flow shear stress application. It should be clearly indicated in the materials and methods whether the application of a fluid flow is used to pre-condition endothelial cells, to seed extravasating cells or for any other purpose. Most of the times, this information is present throughout the text, but not clearly reported in the methodological section. Additionally, when fluid flow is applied, it would be very useful to indicate the levels of flow shear stress (in dyn/cm^2^ or Pa), to allow the comparison among different studies independently from the size of the vascular channel.

Overall, our analysis of the results obtained using advanced 3D models for studying immune and cancer cell extravasation, highlighted differences and similarities between the two processes. The use of microfluidic models allowed distinguishing between direct and endothelium-mediated effects on extravasation. As an example, it has been evidenced that shear stress application reduced endothelial permeability, leading to a decrease in extravasation, whilst inflammatory conditions increased permeability, eventually reflecting in an increased ability to extravasate of both immune and cancer cells. Concerning the direct effects of biophysical stimulation, both immune and cancer cells preferably adhered and transmigrated through the endothelium in lower shear conditions. Although shear stress values were not perfectly matched with the physiological ones, they still allowed evidencing a negative correlation between cell adhesion/TEM and shear stress, which is corroborated by *in vivo* data both on cancer (Follain et al., 2018) and immune cells (Yang et al., 2018). Similar to the correlation between extravasation and shear stress, also chemoattractants showed an analog effect on the extravasation of immune and cancer cells. In particular, leukocytes extravasated only in the presence of specific chemotactic stimuli, as reported *in vivo* (Mitroulis et al., 2015), and the presence of specific chemotactic factors facilitated cancer cell extravasation (Wendel et al., 2012; Borsig et al., 2014), although it was not strictly required to induce the phenomenon. In this context, microfluidic extravasation models were exploited to identify strategies to block the effect of chemoattractants on cancer cells, with the final goal of finding a strategy to stop the metastatization process. A factor differently affecting cancer and immune cell extravasation is represented by the presence of a specific extravascular tissue. Results from microfluidic devices incorporating tissue-like microenvironments showed that the presence of tissue-specific cells plays a relevant role in the metastasis formation, as already hypothesized in the past (Paget, 1989). On the other side, leukocyte extravasation follows a cascade common to most of the human tissues (Maas et al., 2018), even if differences in EVM mechanical properties and endothelium structure have been found to affect leukocyte migration in the tissue. Microfluidic devices proved particularly suitable also to investigate the effects of intrinsic cell properties and of their interactions. As an example, it was possible to visualize the mechanism through which cancer and immune cells cross the endothelial barrier, showing how cancer cells damage the endothelium integrity, and a similar but temporary modification of endothelial junctions was seen also in extravasating leukocytes (Strell and Entschladen, 2008; Sokeland and Schumacher, 2019). Taken together, all these results highlight how cancer and immune cell extravasation shows a similar susceptibility toward factors including shear stress, presence of inflammation, or chemoattractants, whilst immune cell, as opposed to cancer cell, extravasation seems not to be so influenced by tissue-specific factors such as cells included in the 3D matrix.

As described, the majority of these models derives results and takes into account only the effects of single stimuli or specific combinations of few variables, despite the complexity of this physiologically dynamic scenario. Differences and commonalities in immune and cancer cell extravasation emerging from these first uses of microfluidic devices could help in elaborating a common model of the process. The construction of such a model requires the integration of further elements, bringing forward the reproduction of the *in vivo* environment, while maintaining the advantages of a controlled and easy-to-monitor *in vitro* system. The ideal model should hence be able to mimic in a single, detailed vascularized microenvironment all the different aspects of the extravasation process independently from the considered cells. Indeed, ideally only by changing the extravascular environment (tissue-specific cells and EVM) and the extravasating cells, the system would allow a multifaceted analysis of the extravasation process within several different human tissues. Increasing the level of detail and enriching the microfluidic models with more and more elements will lead to a better understanding of this complex process, in view of the development of therapeutic strategies counteracting or enhancing the extravasation of the desired cell type.

## Data Availability Statement

The original contributions presented in the study are included in the article/Supplementary Material, further inquiries can be directed to the corresponding author/s.

## Author Contributions

CA, MM, and SL conceived and designed the study. CM and MC conducted the literature search and screened for eligibility the literature records. CM, MC, and SL conducted the data extraction. CM and SL performed the quality and risk of bias assessment. CM, MC, CA, and SL wrote the manuscript. MM revised the manuscript and supervised the discussion on result organization and presentation. All authors contributed to the article and approved the submitted version.

## Conflict of Interest

The authors declare that the research was conducted in the absence of any commercial or financial relationships that could be construed as a potential conflict of interest.
