# Supplementary material for: Advanced Microfluidic Models of Cancer and Immune Cell Extravasation: A Systematic Review of the Literature
Source: Front Bioeng Biotechnol. 2020 Aug 26;8:907. doi: 10.3389/fbioe.2020.00907 (PMC7479057; doi:10.3389/fbioe.2020.00907)
Supplement: Supplementary file 1 [file Data_Sheet_1.pdf]

Supplementary Materials

Appendix S1. Search strategy

MeSH terms used included:

1. microfluidic* [TIAB] OR microscale* [TIAB] OR micro-scale* [TIAB] OR organ-on-a-chip [TIAB] OR organ-on-chip [TIAB] OR micro-physiological [TIAB] OR microphysiological [TIAB])
2. extravasation [TIAB] OR transendothelial migration [TIAB] OR trans-endothelial migration [TIAB]
3. cancer cell* [TIAB] OR tumor cell* [TIAB] OR “tumour cell* [TIAB] OR metastatic cell* [TIAB] OR metastasis [TIAB] OR leukocyte* [TIAB] OR monocyte* [TIAB] OR eosinophil* [TIAB] OR basophil* [TIAB] OR lymphocyte* [TIAB] OR neutrophil* [TIAB] OR “immune cell* [TIAB]
4. 1 AND 2 AND 3
